# AI-based detection of Certas Plus shunt valve settings in CT scans

**DOI:** 10.1038/s41598-026-45388-2

**Published:** 2026-03-21

**Authors:** Pierre Scheffler, Mukesch Shah, Ramy Amirah, Shahan Momjian, Jürgen Beck, Amir El Rahal

**Affiliations:** 1 Department of Neurosurgery, Medical Center, University of Freiburg, Breisacher Str. 64, 79106 Freiburg im Breisgau, Germany; 2 Faculty of Medicine, University of Geneva, Geneva, Switzerland

**Keywords:** Artificial intelligence, Hydrocephalus, Programmable shunt valve, Computational biology and bioinformatics, Engineering, Health care, Medical research, Neurology

## Abstract

Adjustable pressure cerebrospinal fluid (CSF) shunt valves are widely used in the treatment of hydrocephalus. CSF shunt dysfunctions can manifest with diverse symptoms, often requiring further diagnostic evaluation. Head computed tomography (CT) is frequently used as an initial diagnostic tool. Accurate identification of the current shunt valve setting is crucial for patient management; however, interpretation on CT is difficult due to three-dimensional imaging, metal artefacts, and limited spatial resolution. We therefore developed an artificial intelligence (AI)-based model to automatically assess shunt valve settings in CT scans. We collected 391 head CT scans from patients with CSF shunts featuring a Certas Plus valve. Shunt settings were extracted from medical records and verified on imaging. A 3D U-Net was trained to segment radiopaque valve components, from which the valve setting was inferred. The model successfully segmented valve components in 97.3% of test cases and correctly predicted the exact or an adjacent setting in 96% of cases. The segmentations enable clinicians to interpret and verify the prediction. Our study demonstrates the feasibility of AI-based detection of programmable shunt valve settings in CT scans. The proposed model reliably identifies Certas Plus valve settings and holds promise as a clinical support tool in shunt diagnostics.

## Introduction

Hydrocephalus is a common condition with an overall prevalence of 88/100 000 people^1^. For many forms of hydrocephalus, cerebrospinal fluid (CSF) shunt implantation is one of the main treatment modalities^2^. CSF shunt malfunctions are frequent and can manifest in various ways, leading to frequent diagnostic evaluations in shunted patients. Given the widespread availability of CT imaging across hospitals, even those lacking specialized neurosurgical units, it has become the initial diagnostic tool for evaluating suspected shunt dysfunctions^3^.

In recent years, the use of adjustable pressure shunt valves in hydrocephalus patients has become more prevalent, as they allow adjustment of CSF drainage according to patient symptoms and drainage-related complications^4,5^. Accurately identifying the current shunt valve setting is essential in determining if a shunt malfunction is present. Changes in shunt valve setting can occur unintentionally, e.g., after exposure to strong magnetic fields, such as those generated by MRI machines. These setting changes can then lead to symptoms of over- or underdrainage. However, due to metal artefacts and lower resolution compared to 2D x-ray imaging, assessing the shunt setting on a CT scan can be challenging^6^.

Automated image recognition has made significant advancements in recent years, primarily due to the rise of artificial intelligence (AI)-based models. Convolutional neural networks were the first model architecture to match or surpass human evaluation in image-based classification tasks such as the ImageNet benchmark^7^. They use convolution operations between layers of neurons that resemble the receptive field of neurons in the visual system of animals. Convolutional neural networks have already found various applications in medical imaging tasks such as tumour detection in X-rays, segmentation in CT imaging, or classification of histopathological imaging^8^. For image segmentation AI models, one of the most widely used and effective models is the CNN-based U-Net, which uses convolution operations for image downsampling and subsequent upsampling, resulting in the characteristic U-shaped architecture.

As a result, automated extraction of shunt settings from segmentations of CT scans may be feasible using state-of-the-art AI models, potentially leading to reduced radiation exposure and healthcare costs due to lower neurosurgical referral rates. We therefore chose to assess the feasibility of such an approach in patients with a Codman Certas Plus shunt valve, an adjustable valve featuring a large, radio-opaque setting indicator that is easily read in x-ray imaging.

## Methods

### Data acquisition

We retrospectively screened all CT head imaging of patients with CSF shunts at our hospital from 2017 to 2024. Images were acquired on various CT scanners; the majority were obtained on 2 machines in our neuroradiology department (Siemens SOMATOM Definition AS and Siemens SOMATOM Scope). We collected all images of patients with Certas Plus valves, ensuring a resolution of at least 1 mm in each dimension and the absence of significant artefacts (e.g., movement artefacts at the valve level). The valve was located in a retroauricular position for all images. The shunt setting at the time of imaging was extracted from electronic medical records (which were determined using the Codman Certas Tool Kit) and verified in CT imaging by two experienced neurosurgeons (PS and AER). To ensure strict ground-truth labels for the AI model, images were only included in the dataset if the documented setting matched the one determined from imaging.

### Processing

**Fig. 1:**
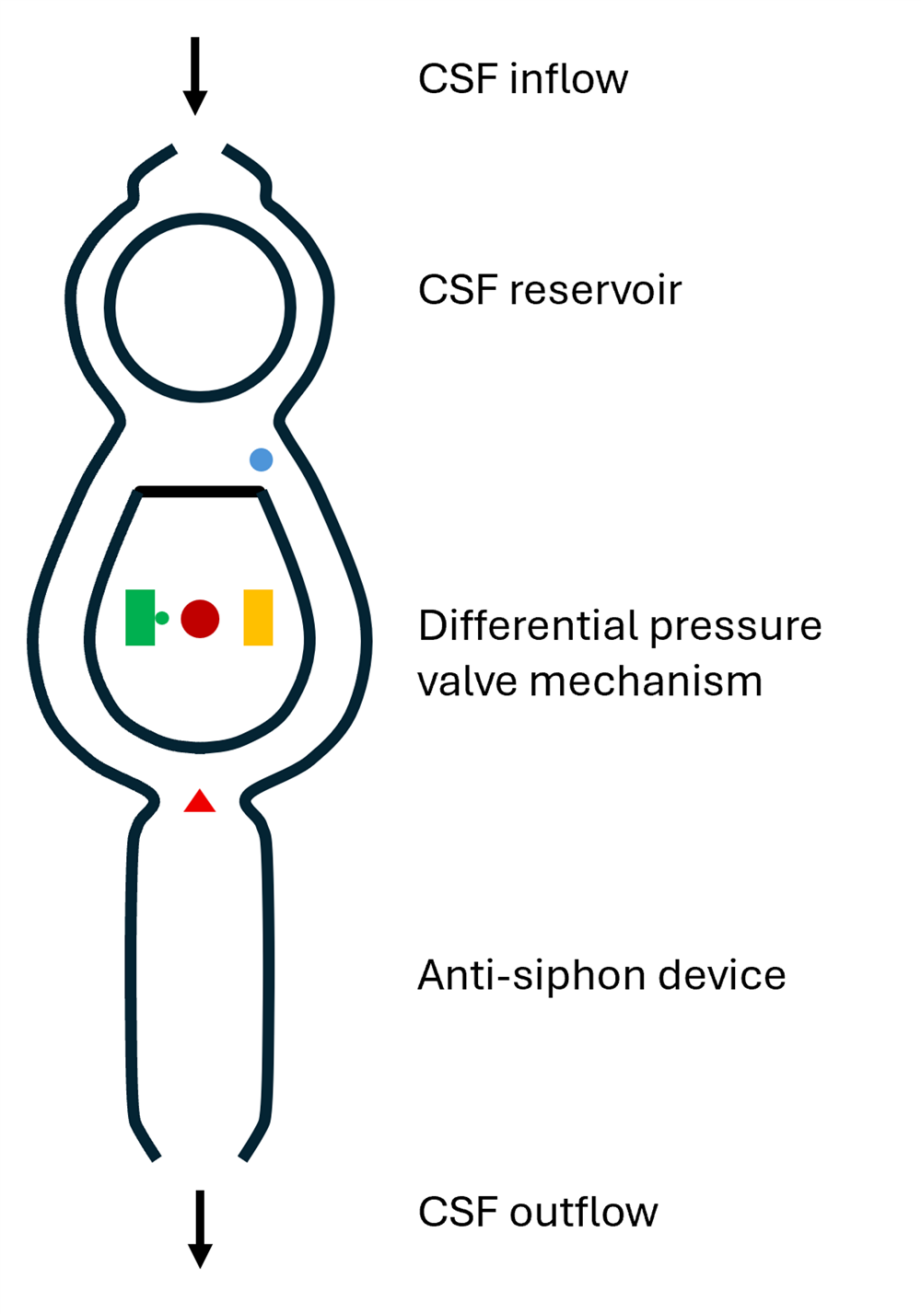
Schematic of a Certas Plus valve with an integrated anti-siphon device, with the 5 segmented radio-opaque markers color-coded as follows: green - magnet with tantalum ball; yellow - magnet without tantalum ball; dark red - rotating construct; blue – right-hand side marker; bright red - distal tip.

After acquiring imaging and shunt setting labels, data were grouped by patient and randomly split into a train and a test dataset with an 80:20 split. Imaging was converted from DICOM to NIfTI using MRIcroGL^9^. We used 3D Slicer to manually segment 5 radioopaque regions for each shunt valve^10^, as illustrated in Fig. 1: the magnet with the tantalum ball (the setting indicator), the magnet without the tantalum ball, the rotating construct, the right-hand side marker, and the distal tip of the shunt valve.

We used nnUNet to train a 3D U-Net to segment the 5 aforementioned markers separately^11^. nnUNet is a framework that automatically adjusts U-Net training parameters to the dataset to optimize model performance without manual intervention. We used the medium-size residual encoder preset for processing full-resolution images. No further adjustments were made to the framework.

**Fig. 2:**
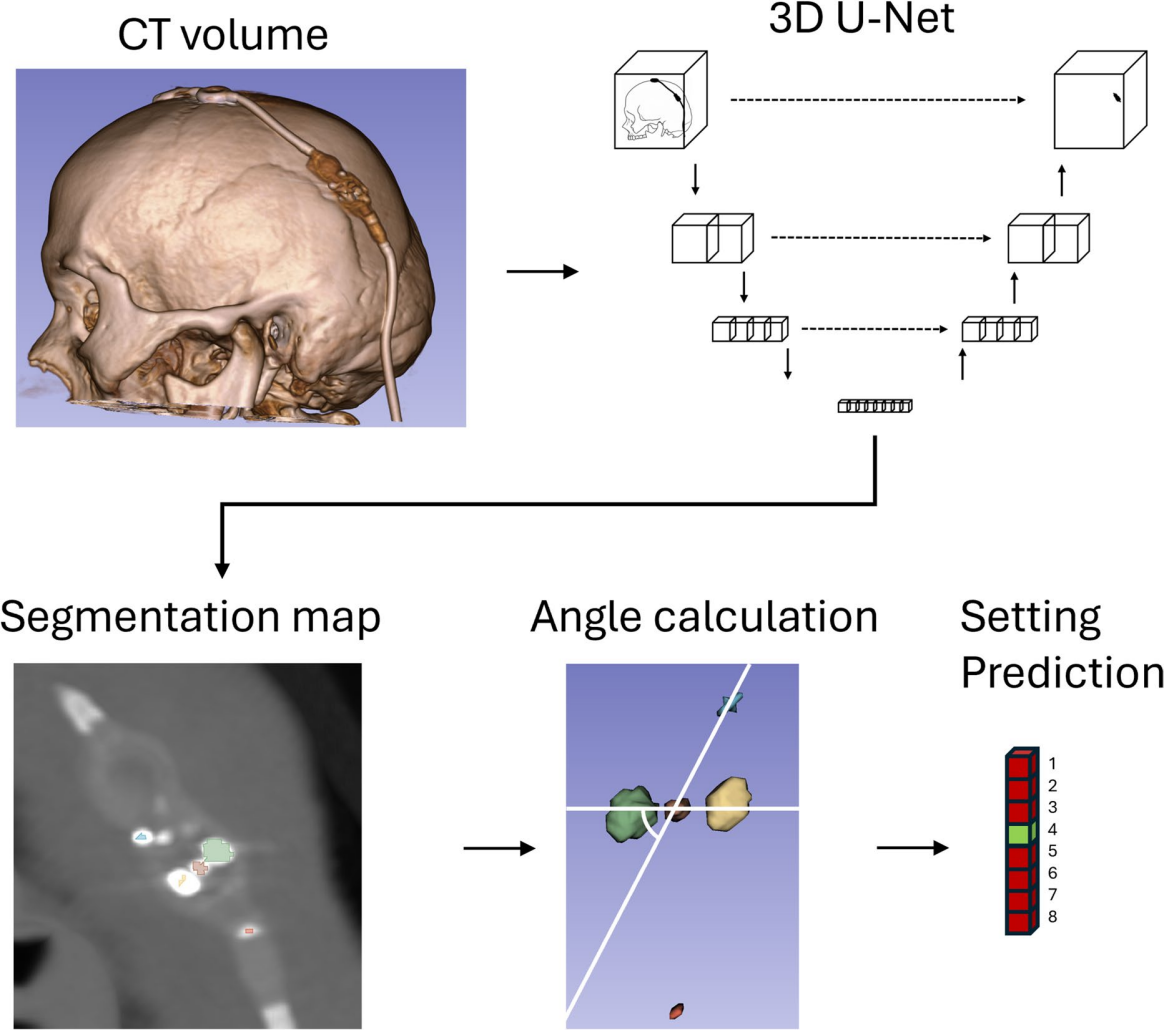
Illustration of the steps for valve setting detection. The full-resolution CT volume is processed by a 3D U-Net to create a segmentation map that labels the radioopaque markers. The centers of mass of these markers are used to calculate the angle of the setting indicator with respect to the right-hand side marker, which is then converted to the shunt setting.

Automated image processing is based on custom code written in Python and performed as illustrated in Fig. 2. First, the CT volume is processed by the U-Net to create a segmentation map of the markers. Then, the center of mass is calculated for each marker. The plane and orientation of the shunt valve are determined based on the rotating construct, the right-hand side marker, and the distal tip of the valve. Then, we calculate the angle between a straight line going through the rotating construct and the right-hand side marker, and a straight line going through both magnets. The angle is then converted to a shunt setting. Based on illustrations provided by the manufacturer, we determined that an angle of 0 degrees (when both axes are superimposed) represents the cutoff between settings 2 and 3. We then attributed zones with a size of 45 degrees to represent each of the 8 consecutive settings, increasing in clockwise order and covering the whole 360-degree range. Test set evaluation was also performed with code written in Python; graphs were generated with the Matplotlib library^12^. Confidence intervals (CI) were calculated using the Clopper-Pearson exact method.

## Results

### Description of dataset

**Fig. 3:**
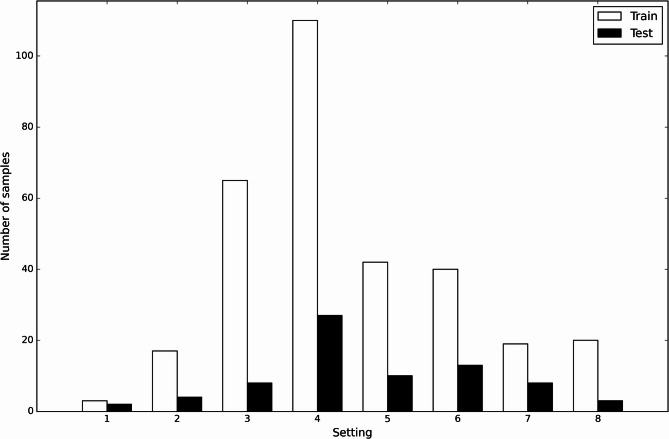
Histogram of the settings associated with all CT images in the train and test datasets, respectively.

Out of 533 initially collected CT scans, clinical documentation regarding the valve setting was available in 486 cases. Manual segmentation and measurement matched the clinical documentation in 391 of these cases, resulting in an initial agreement rate of 80.5%. Out of these 391 scans, 316 were used for model training, and 75 were used for model testing. Figure 3 shows histograms of the distribution of shunt settings per dataset. Train and test datasets show similar distribution, with setting 4 being the most frequent in both groups.

### Setting indicator segmentation and detection

**Fig. 4:**
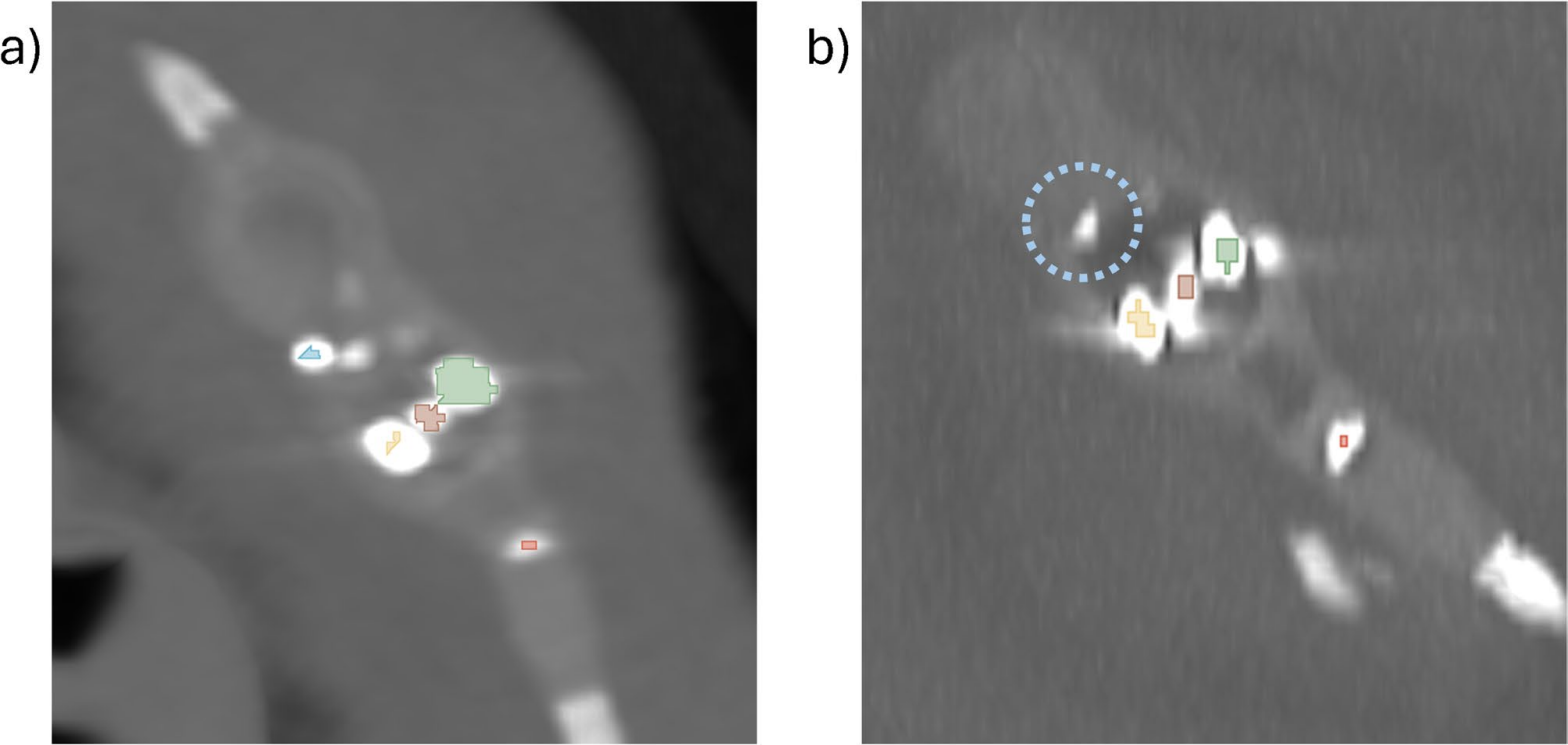
Examples of segmentations of valve setting indicators. (**a**) Valve for which all elements were successfully segmented. (**b**) Valve for which the right-hand side marker could not be detected (the location of the right-hand side marker is circled with a dotted blue line). Colors: green - magnet with tantalum ball; yellow - magnet without tantalum ball; dark red - rotating construct; blue – right-hand side marker; bright red - distal tip.

Our AI model was able to determine the angle of the setting indicator in 73 out of 75 (97.3%) of all test cases. Results for the Dice similarity coefficient (DSC) and intersection overunion (IoU) are summarized in Table 1. In cases where the setting could not be determined, it was because the model could not detect the right-hand side marker, as demonstrated in Fig. 4.

**Table 1:** Results for the Dice similarity coefficient (DSC) and intersection over union (IoU) for each of the segmented areas in the test dataset expressed as mean and standard deviation (SD).

Predicted label	Mean DSC	SD DSC	Mean IoU	SD IoU
Magnet with tantalum ball	0.841	0.129	0.741	0.146
Magnet without tantalum ball	0.773	0.160	0.651	0.165
Rotating construct	0.721	0.159	0.585	0.172
Right-hand side marker	0.631	0.221	0.496	0.225
Distal tip	0.386	0.302	0.290	0.280

### Angle of valve setting indicator

**Fig. 5:**
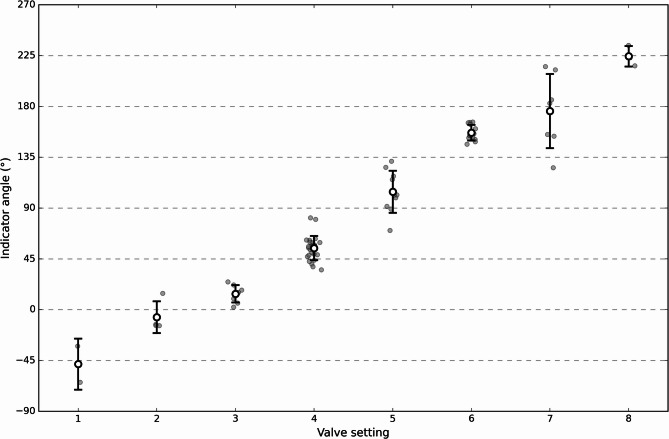
Illustration of the indicator angle determined by the model in relation to the shunt valve setting. Grey dots indicate individual sample values; white dots indicate mean values; standard deviations are represented by error bars. The dotted horizontal lines represent the angle cutoffs for the discrete settings as detailed in the methods section.

Figure 5 shows the indicator angle determined by the model in relation to the shunt setting; the numerical values of the mean angles, their standard deviation, and the expected angle per setting are shown in Table 2. Mean angles increased progressively with each setting. The expected angle is within 1 standard deviation for settings 1, 5, 6, and 7, and within 2 standard deviations for settings 2, 3, 4 and 8. The standard deviations overlap for settings 2 and 3, and 6 and 7, respectively.

**Table 2:** Overview of measured angles per setting with mean and standard deviation, and expected angle per setting.

Setting	Mean (°)	Standard deviation (°)	Expected angle (°)
1	-48.3	22.6	-67.5
2	-6.7	14.1	-22.5
3	13.9	7.7	22.5
4	54.5	10.8	67.5
5	104.4	18.6	112.5
6	156.7	6.9	157.5
7	175.9	32.9	202.5
8	224.4	9.1	247.5

### Confusion matrix for valve settings

**Fig. 6:**
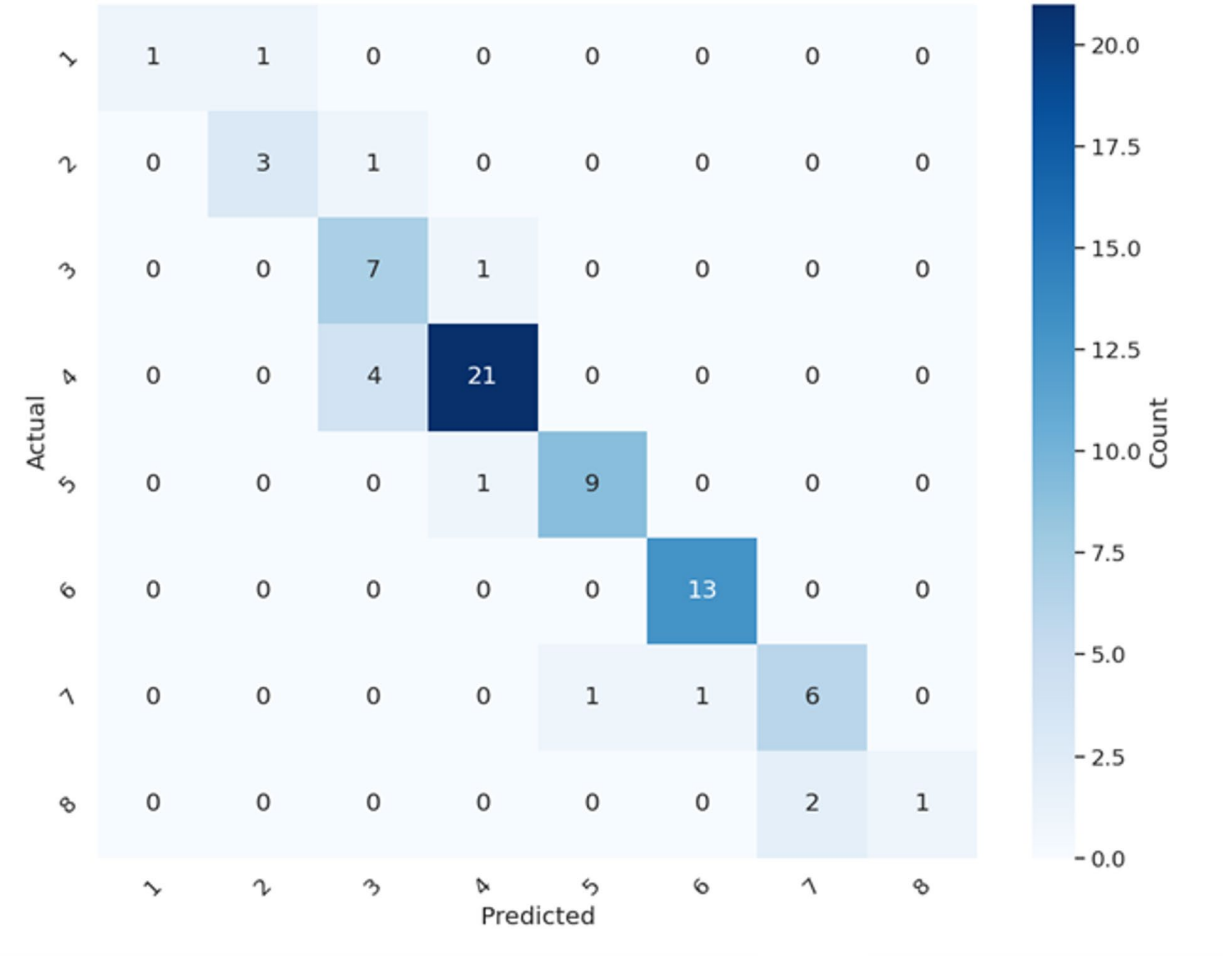
Confusion matrix of our model comparing the true setting (as determined by the Certas Tool Kit and verified in imaging) to the setting determined by the model.

Figure 6 shows the confusion matrix for all the shunt settings. Most settings are correctly identified; the settings that are not correctly determined are mostly identified as settings adjacent to the real setting. Setting 4 for example, the most common setting, is predicted as setting 3 or 5 in 8.7% of cases, but never as 1, 2, 6, 7, or 8. Overall accuracy was 81.3% (CI: 70.7% − 89.4%); accuracy increases to 96% (CI: 88.8% − 99.2%) if prediction of adjacent settings is considered acceptable.

## Discussion

This study demonstrates that AI-assisted identification of shunt settings in CT scans is feasible. Our segmentation model was able to correctly predict the documented setting in 81.3% of cases and the documented or an adjacent setting in 96% of cases, which may be considered clinically useful.

Hydrocephalus and CSF shunt malfunctions are common and result in substantial healthcare costs: Shunt-related costs exceed a billion dollars per year in the United States alone^13^. One of the driving cost factors are interfacility transfers for extended diagnostics^14^. Verification of the shunt setting is one of the many possible reasons for such a transfer. Although setting verification in CT scans by humans is technically possible and has been described in previous publications using multi-planar reconstruction, this requires skill and is limited by CT resolution and image artefacts that usually require metal artefact reduction algorithms^6,15^. As demonstrated during our data collection phase, manual evaluation by experienced neurosurgeons matched the available clinical documentation in 80.5% of cases, which represents a lower-bound for human baseline accuracy in a real-world clinical setting. In this context, our AI model’s exact accuracy of 81.3% is competitive with this human baseline. AI-assisted CT interpretation, including shunt setting detection, could reduce unnecessary transfers in patients with unremarkable scans and correctly set shunt valves, thereby lowering healthcare costs.

**Fig. 7:**
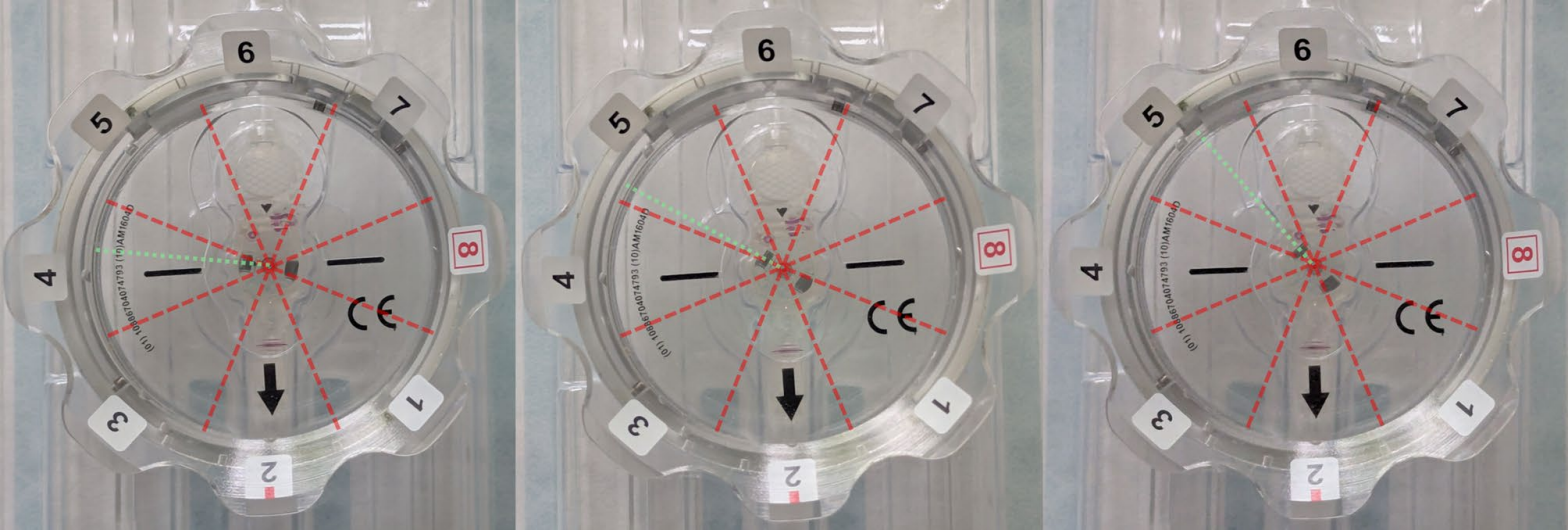
Possible settings of the Codman Certas Plus valve. The valve can be set in position 4 (left) or position 5 (right), as well as in any intermediate position between these settings (middle). As a result, adjacent settings cannot always be distinguished. The red dashed lines represent limits between settings, the green dotted lines represent the position of the indicator.

Concerning overall model prediction accuracy, in our analysis, we show that in most incorrectly predicted cases, our model predicts a valve setting that is directly adjacent to the documented setting. We believe that this is due to a design limitation of the Certas valve. Although the valve is marketed as having discrete pressure settings in a range from 1 to 8, the rotating valve setting indicator can be set continuously, as demonstrated in Fig. 7. This is reflected in our model’s measured indicator angles, which varied continuously rather than clustering around eight discrete values. As a result, the discrete setting attributed to some valves can be ambiguous. Metal artefacts can cause a slight deviation of the measured angle from the axis determined by the magnet-based toolkit, leading to discrepancies between settings measured in the image and with the Certas Plus Toolkit.

Given that adjacent settings have similar flow resistance, confusion between adjacent settings may not be clinically relevant. The manufacturer documentation states that Certas Plus valve pressure settings have tolerances between 20 and 35 mmH_2_O depending on the setting, resulting in significant possible overlap in outflow resistance between adjacent settings. If we regard predictions of settings adjacent to the documented setting as correct, we achieve an overall accuracy of 96% and a useful setting prediction for 72 out of our 75 test cases. The only relevant distinction between adjacent settings that matters from a clinical perspective is the distinction between setting 1 (very low outflow resistance) and setting 8 (functionally closed valve). In our test dataset, no confusion occurred between these 2 settings, and no ambiguous indicator angles were measured. However, given the scarcity of these 2 settings in our dataset, we cannot rule out the possibility of confusion between them. We see this as a limitation of the valve itself; such confusion could also occur when setting the valve using the official Certas Plus Tool Kit or during dedicated shunt valve X-ray imaging.

In 2 of the 3 remaining test cases, the model did not return an angle, thereby giving the clinician the option to manually verify the shunt setting based on the segmentations and the original image. Previous studies have explored similar applications of AI-based image analysis and achieved comparable results. For example, identification of valve types in X-rays with convolutional neural networks and reached accuracies of 95–99%^16–19^.

For the test cases in which the model failed, no specific reason, such as strong image artefacts or unusual valve positioning, could be identified. The main issue with our model was that it was unable to segment all the indicators required for angle calculation. A known limitation of models trained with nnUNet is that they have difficulty segmenting tiny structures in large volumes^11,20^. As a result, the right-hand side marker and the distal tip of the shunt valve, which only occupy a few voxels in CT imaging, can be missed, making angle calculation impossible. Future models using different training methods could improve upon this model.

The difficulty of segmenting smaller structures is also reflected in the DSC and IoU metrics. Compared to U-Net performance in other medical image segmentation benchmarks, performance is relatively low, and continuously decreases with the size of the segmented structure^21^. Aside from model architecture limitations, this is also due to the relative increase in volume of the surrounding metal artefact for smaller structures, as can be seen in Fig. 4. This reduces the segmentation quality of both the U-Net and the manual ground-truth segmentations. However, the low DSC and IoU metrics are of limited relevance to the final setting prediction, because only large segmentation errors would have a relevant effect on the calculated center of mass for the individual markers that would then significantly alter the setting indicator angle calculation.

Another issue we anticipated when evaluating the settings of Certas models is the potential confusion between opposing settings. This is because the rotating part of the setting indicator is almost symmetrical, except for the sub-millimeter-sized tantalum ball on one of the magnets. However, the segmentation tool was able to reliably identify the correct orientation of the settings indicator in all test cases. Although the Certas valve indicator has various design shortcomings that should limit the setting prediction quality in CT scans by AI models, we show that automated valve setting detection is feasible and achieves clinically useful performance.

The main limitation of our study is that our model was trained and evaluated on a dataset retrospectively collected from a single neurosurgical center, with images mostly acquired on two devices from the same manufacturer. Further studies based on prospectively collected multi-center datasets will be necessary to confirm that our model performs well irrespective of CT scanner type and image postprocessing. In addition, valve setting prediction in our model is currently limited to patients with a single Certas Plus valve. It remains to be seen whether this approach can be extended to multiple valves or other valve types in the future.

## Conclusions

This study provides the first demonstration of AI-based detection of programmable shunt valve settings in CT imaging. Using a 3D U-Net segmentation approach, our model achieved clinically relevant accuracy, correctly predicting the documented setting or an adjacent setting in 96% of cases. Future work should validate these findings in larger, multicenter datasets and explore extension to other valve types. Ultimately, automated valve setting identification could reduce reliance on manual setting verification and dedicated shunt valve X-ray imaging.

## Data Availability

The training and test datasets are not publicly shared due to privacy concerns. Parts of the datasets containing data from patients who agreed to share it with other researchers will be made available upon reasonable request to the corresponding author.

## Abbreviations

AI: Artificial intelligence
CSF: Cerebrospinal fluid
CT: Computed tomography
